# Changes in use of time, activity patterns, and health and wellbeing across retirement: design and methods of the life after work study

**DOI:** 10.1186/1471-2458-13-952

**Published:** 2013-10-10

**Authors:** Carol A Maher, Nicola W Burton, Jannique GZ van Uffelen, Wendy J Brown, Judy A Sprod, Tim S Olds

**Affiliations:** 1 University of South Australia, Health and Use of Time Group, Adelaide, South Australia, Australia; 2 The University of Queensland, School of Human Movement Studies, Brisbane, QLD, Australia; 3 Victoria University, Institute of Sport, Exercise and Active Living, Melbourne, VIC, Australia

**Keywords:** Ageing, Retirement, Use of time, Physical activity, Sedentary behaviour, Social interaction, Longitudinal, Wellbeing, Exercise, Life events

## Abstract

**Background:**

Retirement is a major life transition during which people restructure everyday activities; however little is known about this. The primary aim of the Life After Work study is to comprehensively measure changes in time use and patterns of physical activity and sedentary behaviour, and its associations with health and wellbeing, across the retirement transition.

**Methods/Design:**

A target sample of 120 participants aged 50 years and over will be recruited in two Australian state capital cities, Adelaide and Brisbane. Participants will undertake a battery of assessments approximately 3 months prior to retirement, and 3, 6 and 12 months post-retirement. Measures will include self-reported use of time (using the Multimedia Activity Recall for Children and Adults), objectively assessed physical activity and sedentary behaviour (using Actigraph GT3X+ accelerometers), self-reported health and well-being (using a battery of questionnaires including the Short-Form Health Survey (SF-36), Australian Unity Personal Well-being Index (AUPWI), Depression Anxiety Stress Scales 21 (DASS21), Short Warwick-Edinburgh Mental Well-being Scale, UCLA Loneliness Scale, Rosenberg Self-Esteem Scale), retirement circumstances and socio-demographic characteristics, objectively assessed anthropometric measures (height, weight and waist circumference), and resting blood pressure. Multivariate mixed models will be used to examine changes in use of time, health and well-being across retirement.

**Discussion:**

The results will provide important new information that will inform the development of lifestyle and policy interventions to address and improve health and well-being in retirement.

## Background

The Australian population is ageing, and it is estimated that the proportion of the population aged 65+ years will nearly double from 13% to 24% in the next 40 years [1]. About 65% of people aged 15+ years participate in the workforce [2], with most employees retiring at mean age of 53 years [3]. As the baby boomer generation ages, the proportion of retirees is increasing. Furthermore, the length of time spent in retirement is increasing as a result of increasing life expectancy [4]. Retirees are therefore an increasingly important demographic sector, especially in regards to health, since the burden of disease is disproportionately incurred in older people [5].

Retirement is a major life transition, and a point at which time use and activity patterns, as well as social interactions, leisure pursuits, and other daily behaviours, are restructured. However, changes in daily activity patterns, social interaction and enjoyment of activities, across retirement are not well understood. Some evidence suggests that leisure time physical activity may increase in middle-to-upper socioeconomic status groups [6-10], but that overall physical activity levels decline, due to lost occupational and associated physical activity (e.g. active transport to work) [6,11,12]. Although retirement might be associated with a decline in occupational sitting time, especially among those who retire from desk-based jobs, not much is known about changes in sitting time in other life domains. A small number of studies have found that self-reported television viewing time increases across retirement [8,9,13], but changes in other behaviours, such as computer use and general sedentary leisure (e.g., reading), or objectively-measured sedentary time overall, are poorly understood. Furthermore, very little is known about how other attributes of activities, such as social interaction and enjoyment, change across retirement.

Given the established links with cardiometabolic health, changes in physical activity and sedentary behaviour may have implications for health and wellbeing in retirement. Restructuring of use of time at retirement, may also have flow-on effects, which can affect health in complex ways. For example, when people undertake a new leisure-time physical activity, they may reduce physical activity in other areas, such as walking or gardening, which may lead to no net health gain. In contrast, they may reduce their sedentary behaviours, such as television viewing, which could amplify the health benefits. Furthermore, they may reduce sleep, or spend less time in intellectual activities such as reading, or in social activities, with ensuing health deficits. This study will capture such ripple effects of changes in daily activity patterns by means of a use-of-time methodology. The use-of-time approach systematically records daily activities across a broad variety of life domains, rather than capturing changes in a single domain. Thus, this study sets out to comprehensively capture changes in use of time and its associations with health and well-being across the retirement transition.

## Methods/Design

### Aim

The Life After Work study aims to describe changes in time-use across retirement, and associations with health and well-being, with a particular focus on physical activity, sedentary behaviours, sleep, social interaction, and enjoyment.

### Study design

Life After Work is a multi-site longitudinal cohort study with data collection at four time points; at baseline prior to retirement, and three post-retirement follow-up assessments, at 3, 6 and 12 months after retirement.

### Study sample

The aim is to enrol 60 participants at each site, for a total of 120 participants.

Participants are being recruited from two Australian state-capital cities, Adelaide and Brisbane. The eligibility criteria are outlined in Table 1. Given that the retirement process varies widely between individuals, the eligibility criteria are designed to accommodate people with a variety of retirement situations. In particular, it is worth noting that part-time work status is permissible in the pre- and post-retirement periods. In order to assess changes in time use related with retiring, the decrease in working hours over retirement must be at least 19 hours, with no more than 11 hrs/week of paid employment after retirement.

**Table 1:** Eligibility criteria for the life after work study

**Inclusion criteria**	**Exclusion criterion**
1. Participant self-identifies as intending to retire within 6 months.	1. Presence of a major (life-threatening) medical condition or undergone major treatment in the last 6 months.
2. Aged over 50 years.	
	2. Intending to relocate away from the data collection site (Adelaide or Brisbane) within the 12 month follow-up period.
3. Pre-retirement ≥19 hours per week in paid employment (i.e. working half-time or more).	
4. Anticipated post-retirement workload:	
a) ≤11 hours per week paid employment AND	
b) Paid employment will reduce by ≥19 hours	
5. Able to speak and write in English.	

Recruitment is taking place on a rolling basis from May 2012 to July 2013. A variety of recruitment avenues are being utilised for both sites, including commercial recruitment agencies, emails/advertising in large workplaces, media articles in major and community newspapers/radio stations, promotion via municipal councils and special interest groups, such as superannuation retirement sessions.

### Protocol

For each participant, data collection is occurring at four time points over a 13–18 month period. The first and final (4^th^) assessments are face-to-face, while assessments 2 and 3 are undertaken remotely, using mail/telephone/online surveys. A summary of the outcome measures at the various time points is provided in Table 2.

**Table 2:** Outcome measures at each time point

	**Baseline**	**Follow-up 1**	**Follow-up 2**	**Follow-up 3**
Timing	2-6 months prior to retirement	2-4 months after retirement	5-7 months after retirement	11-13 months after retirement
Assessment mode	face-to-face	remote	remote	face-to-face
Use of time (MARCA)	**✓**	**✓**	**✓**	**✓**
Activity enjoyment & social interaction (MARCA)	**✓**	**✓**	**✓**	**✓**
Objective activity (accelerometry)	**✓**	**✓**	**✓**	**✓**
Well-being (questionnaire)	**✓**	**✓**	**✓**	**✓**
Sociodemographics & retirement circumstances (questionnaire)	**✓**	**✓**	**✓**	**✓**
Self-reported health (questionnaire)	**✓**	**✓**	**✓**	**✓**
Objective physical health (anthropometry & blood pressure)	**✓**			**✓**

Key: MARCA= Multimedia Activity Recall for Children and Adults.

### Outcome measures

#### Use of time, enjoyment and social interaction

Use of time, activity enjoyment and social interaction is being measured via telephone interviews using the Multimedia Activity Recall for Children and Adults (MARCA). This is a computerised 24-hour use-of-time recall tool, which is being administered at all four study time points. The MARCA captures use of time by asking participants to recall their previous day from midnight to midnight, using meal times as anchor points. Participants recall their day in time slices of five minutes or more by choosing from 520 different activities. The MARCA is also able to capture enjoyment and social interaction. Participants are asked to rate their enjoyment level for all activities, on a scale of 0 (“I hated it”) to 10 (“I loved it”), and who they are with during activities, using the categories of “alone”, “spouse/partner”, “kids”, “friends/other adults”, “parents” and “others”. For analysis, individual activities can be collapsed into key use-of-time domains (physical activity, television time, transport, quiet time, self-care, socio-cultural, work/study, chores, sleep and screen time), allowing daily duration and energy expended in various activity domains to be calculated. Each activity in the MARCA can also be assigned a metabolic cost (MET value) based on an expanded version of the Ainsworth compendium of physical activities [14,15], making it possible to calculate energy expenditure variables, such as daily Physical Activity Level (PAL) which is the time-weighted average of MET values over the day [16]. The MARCA has excellent test-retest reliability in adults (0.990-0.997; p≤0.0001) for MVPA, PAL, sleep and screen time), and strong convergent validity with accelerometry (rho = 0.72) [16]. Furthermore, a recent comparison with the gold standard doubly labeled water showed correlations of rho = 0.70 for total daily energy expenditure [17].

At each time point, the MARCA is being administered on two occasions, each time recalling two consecutive days, resulting in a total of four being recalled days at each time point. At baseline, at least one of the four days must be a work day, and at least one a non-work day, for each participant. In each of the follow-up assessments, the MARCA is being administered on the same days as it was at baseline.

#### Objective physical activity and sedentary behaviour

The Actigraph GT3X+ accelerometer (ActiGraph, Ft. Walton Beach, FL) is being used to objectively monitor physical activity and sedentary behaviour at all four study time points. The accelerometer is worn at the waist on an elasticized belt, on the right mid-axillary line. Participants are requested to use a wear-time log sheet to record time the device is put on, removed, and sleep and nap times. Since other studies have reported problems with meeting minimum-wear-time requirements with a waking hours protocol (i.e. daily instrument removal for sleep), participants are being encouraged to wear the accelerometer 24 hours per day for 7 consecutive days. Accelerometers are initialised using ActiLife software [18], with an epoch length of 1 second and sampling rate of 80 Hz. Accelerometers will be returned to the study site by reply-paid mail, at which time the research team verify the data for completeness using ActiLife software. Ninety minutes of consecutive zeros are being used to detect invalid minutes (i.e. minutes in which the accelerometer was not worn). The minimal amount of accelerometer data that is considered acceptable is 4 days, including at least one weekend day, with at least 10 hours of valid wear time per day. If accelerometer data are incomplete, participants are asked to wear the accelerometer for an additional 7 days (to a maximum of 14 days) to ensure that the minimal data requirements are met. For analysis, the cut offs of ≥2020 to 5999 counts per minute will be used to detect moderate physical activity, ≥6000 counts per minute for vigorous physical activity [19] and <100 counts per minute for sedentary activity [20]. Actigraph accelerometer have moderately-strong validity with reference to doubly labelled water (r = 0.58, p<0.001) [21] and excellent intra- and inter-instrument reliability (coefficient of variation = 2.9% and 3.5% respectively) [22].

#### Mental health and wellbeing

A questionnaire booklet concerning health and well-being is being used at all four time points. At baseline and final follow-up it is being completed in hard copy, and at follow-ups 1 and 2 the questionnaire is being completed in either hard copy, or electronically, at the participants’ discretion. The questionnaire contains numerous previously-developed scales, as follows:

##### Quality of life

The Short-Form Health Survey (SF-36) [23] contains 36 items with Likert scales and dichotomous yes/no response options. Scores are summed to derive eight subscales (physical functioning, role-physical, bodily pain, general health, vitality, social functioning, role-emotional and mental health) and two summary measures (physical health and mental health). Test-retest reliability over a two week interval has been shown to be moderately strong (r=0.60 to 0.81) [23]. Comparisons of four equivalent dimensions of the SF-36 and the Nottingham Health Profile demonstrated evidence of convergent validity, though correlation coefficients varied (r = 0.18 to 0.68) [23].

##### Life satisfaction

The Australian Unity Personal Well-being Index (AUPWI) [24] contains eight questions, measuring satisfaction with life as a whole, standard of living, health, life achievement, personal relationships, perceptions of safety, community connection, future security and spirituality/religion. Responses are collected on a 10 point rating scale ranging from zero (“completely dissatisfied”) to 10 (“completely satisfied”). It has good test-retest reliability (r = 0.84) over a test period of one to two weeks (Lau & Cummins 2005 in [25] and convergent validity with the Satisfaction with Life scale of r = 0.78 [26].

##### Depression and anxiety

The Depression Anxiety Stress Scales (DASS) 21 [27] has seven items in each of the three scales, with respondents using a four point Likert scale to rate the extent to which they have experienced negative emotional states in the past week. Internal consistency has been shown to be excellent (Cronbach’s α = 0.93 (95% CI = 0.93 to 0.94) for the total scale [27]. The scale shows moderate to high convergent and discriminant validity compared to the Personal Disturbance Scale (r = 0.78 for depression and r = 0.72 for anxiety) and the Hospital Anxiety and Depression Scale (r = 0.66 for depression and r = 0.62 for anxiety) [28].

##### Mental well-being

The Short Warwick-Edinburgh Mental Well-being Scale [29] has seven items, with respondents asked to rate the extent to which they have experienced positive feelings and thoughts in the past two weeks on a five-point Likert scale. The correlation between the short and long (14 item) versions of the Warwick-Edinburgh Mental Well-being Scale is excellent (Spearman’s correlation = 0.95) [30]; and the test-retest reliability of the long Warwick-Edinburgh Mental Well-being Scale over a one-week period is high (r = 0.83) and correlates well with the Positive Affective section of the Positive and Negative Affect Schedule (PANAS; r = 0.71) [29].

##### Loneliness

The UCLA Loneliness Scale [31] contains ten positively and ten negatively worded questions, with a four-point Likert scale. Test-retest reliability over a 12 month period is moderately high (r = 0.73) [31] and the scale has been shown to correlate moderately well with other loneliness measures, such as the Social Provisions Scale (−0.54 to −0.68) [31].

##### Self-esteem

The Rosenberg Self-Esteem Scale comprises ten items scored on a four-point Likert scale [32]. Test-retest reliability over a two week period is high (r=0.85-0.88) [32] and the scale has been long considered as a valid measure of self-esteem, on the basis that it has concurrent, predictive and construct validity in known groups; that it correlates significantly with other measures of self-esteem, such as the Coopersmith Self-Esteem Inventory; and that it correlates in the predicted direction with measures of depression and anxiety [32].

#### Retirement circumstances and sociodemographics

Retirement circumstances are being captured using purpose-designed questionnaire items, since no suitable previously-developed tools could be identified. Respondents are being asked to indicate the reasons for retirement from 13 options, including to pursue other interests, sickness, care responsibilities, retrenchment, declining interest in work and stress. Respondents are also asked the extent to which they have made plans for various aspects of life including social, physical and mentally stimulating activities.

Socio-demographic information include age, sex, postcode, language spoken, marital status, household structure, Aboriginal or Torres Strait Islander origin status, occupation, education and income. Items are based on those used in the Australian Longitudinal Study of Women’s Health [33] and in the Australian Census [34].

#### Self-reported health

General health is being assessed using items from the Australian Longitudinal Study of Women’s Health [33]. Items include an overall self-reported rating of general health, medical service utilisation, experience of 14 different symptoms (such as pain, fatigue, sensory problems, breathing difficulties etc.), use of prescribed medications and diagnosis status for nine various health conditions (such as cancer, diabetes, asthma, cardiovascular disease etc.).

#### Objective physical health

##### Anthropometry

Body mass, stature and waist circumferences are being measured at baseline (pre-retirement) and final follow up (12 months post-retirement), according to International Standards for Anthropometric Assessment (ISAK) procedures [35]. Body mass is being measured using portable Tanita HD332 scales (Tanita Corporation of America, Arlington Heights, IL; Adelaide site) and Nuweight Log 842 BMI scales (Newcastle Weighing Services, Newcastle, Australia; Brisbane site) after all outer clothing, heavy pocket items and shoes and socks are removed. Stature is being measured without shoes using an Invicta height measure (Invicta Group, Leicester, UK; Adelaide site) and the Seca 213 portable stadiometer (Seca Ltd, Hamburg, Germany; Brisbane site), with the participant’s head in the Frankfort Plane. Stature will be measured with the participant fully erect with feet together, at the end of a deep inhalation. Waist circumference is being measured using Lufkin W606PM steel girth tape (Cooper Industries, Lexington, SC) at the end of gentle expiration at the narrowest waist level between lower rib margin and the iliac crest when viewed from the front. If this is not apparent it will be taken at the mid-point between the lower rib margin and the iliac crest.

For each measurement, two measures are being obtained, and the average will be used for analysis. A third measurement will be obtained if the first two measurements are greater than 0.5 cm or 0.1 kg apart and the average of the two closest measurements will be used for analysis. The Body Mass Index (BMI; body mass (kg)/height (m^2^)) and waist-to-stature ratio will be calculated using standardised calculations.

##### Resting blood pressure

Blood pressure is being measured using a non-invasive automated sphygmomanometer (Dinamap Pro 100; Critikon, Tampa, FL) at the baseline (pre-retirement) and final (12 months post-retirement) assessments. Blood pressure is beingrecorded with participants seated and rested for 5 minutes, with the measurement repeated after a one minute interval, and the average used for analysis (a third measurement is obtained if the first two measurements differ by greater than 5 mmHg). The Dinamap Pro 100 is recognized to be highly reliable relative to standard mercury sphygmomanometry (r = 0.89 systolic, r = 0.81 diastolic) [36].

### Ethical issues

The overarching LAW protocol has been approved by the University of South Australia Human Research Ethics Committee. The Brisbane site-specific protocol has also been approved by The University of Queensland Behavioural and Social Sciences Ethical Review Committee. Written informed consent is being obtained from all participants.

### Study management

This study is partially funded by an Australian Research Council Project Grant. TO, WB, CM, NB and JVU are the chief investigators on the grant, and have been responsible for development of materials and protocol and overseeing all aspects of the study. JS is a PhD candidate and is coordinating data collection and training of Adelaide personnel. Investigators meet via regular teleconferences to discuss overall study progress, direction and future goals. The University of South Australia is the administering site for the study, and is responsible for development of the Standard Operating Procedures, quality control audits and study administration. The Adelaide and Brisbane sites are individually responsible for hiring personnel and collecting all data elements according to the Life After Work protocol, with the exception of the MARCA telephone interviews, which are being conducted from the Adelaide site.

### Data management and quality control

Data are being single-entered into standardized spread sheets and stored separately at the Adelaide and Brisbane sites. A number of procedures are being used to ensure data completeness and quality. All data collection materials are being quality checked by research personnel on the day of administration, to verify data completeness. Missing or ambiguous items are verified with participants with a follow up telephone call. During data entry, variables are checked for accuracy with assigned range checks. Accelerometry data are being checked for wear time validity using the ActiLife software. The MARCA software incorporates self-checking mechanisms including outlier detection. In addition, 5% of data from the Adelaide and Brisbane sites are audited and checked against source data to ensure accuracy of data entry. Site personnel are informed of data entry error rates, nature of the errors, and any other quality control issues identified. Any data entry errors identified during the audit are corrected immediated, and if necessary, site personnel will be retrained.

### Statistical analysis

Data on participants’ sociodemographic characteristics will be summarised using descriptive statistics.

Random-effects mixed modelling [37] will be used to determine how key variables change across the various data collection points. The predictor variable will be time (pre-retirement, 3, 6 and 12 months post-retirement) and the response variables will include:

•Key use of time domains, i.e. physical activity, screen time, transport, quiet time, self-care, socio-cultural, work/study, chores and sleep (from MARCA recalls).

•Activity enjoyment and social interaction (from MARCA recalls).

•Well-being (quality of life, well-being, depression and anxiety, loneliness and Self-esteem, from questionnaire data).

•Self-reported health ( from questionnaire data)

•Physical health (from physical examination).

Multiple linear regression models will be used to examine the relative influence of pre-retirement characteristics (age, sex, and pre-retirement socio-economic status, mental and physical health, psychosocial and time use patterns) on post-retirement use of time and physical activity. Statistical significance will be defined as p<0.05.

### Sample size calculations

A priori power calculations were performed to calculate the sample size required to detect changes in mean values for select study variables (moderate-to-vigorous physical activity, sedentary behaviour and accelerometer counts) over time. Based on four assessment time points and a power of 80%, a sample size of 104 is required to detect small to medium effect sizes (Cohen’s d = 0.25) with a significance criterion of α = 0.01 to allow for multiple comparisons. To allow for 15% drop-out, a final sample size of 120 is being sought, with a target of 60 participants at each site.

## Discussion

The Life After Work study will track how use of time, daily activity patterns, health and well-being change as people transition into retirement. It is the first study, to our knowledge, to comprehensively assess changes in use of time, including important dimensions such as physical activity, sedentary behaviours, social interaction, sleep and enjoyment, across this major life event.

Developing the eligibility criteria for “retirement” was difficult, given the many forms that retirement can take. The final eligibility criteria are a balance between offering flexibility (to allow inclusion of people who work part- or full-time prior to retirement, and also allow people who undertake a degree of paid work in retirement) while also requiring participants to undergo a sufficient reduction in working hours across retirement to have a foreseeable impact on lifestyle and daily activity patterns. We acknowledge that the eligibility criteria will not allow inclusion of every possible retirement path, particularly if this involves a gradual reduction in working hours over an extended period. Similarly, the eligibility criteria are “duration” based, and will not allow inclusion of retirees who consider themselves to have “retired” if they have taken on a less responsible role, without a considerable reduction in their working hours. Although this could potentially decrease the generalizability of the results to the entire population of people who retire, the flexibility of the definition of retirement employed in this study should improve the representativeness, given that participants who follow a wide variety of retirement paths are being included.

Given the modest financial support for this study, a population-based recruitment strategy is unfortunately not feasible. Rather, recruitment will involve promoting the study widely in work places, municipal councils, in the media and through community organisations. While every effort will be made to recruit a diverse sample, the voluntary nature of participation may impact on the representativeness of the final sample.

Strengths of the study include its multi-site nature, which will improve the generalizability of the study findings over a single-site design. A comprehensive battery of outcome measures is being used to assess both physical and psychosocial health. In particular, the MARCA offers richness of data regarding use of time, social interaction and enjoyment, and is highly reliable and valid. The standardized protocol and quality control procedures will ensure accurate and reliable data. The study combines both self-report and objective measures, and the multiple time points post-retirement will provide rich information about the retirement transition as a process. The study involves a 12 month follow up period, with assessment at approximately 3 months, 6 month and 12 months post retirement, thereby allowing for short term and longer term establishment of new activity patterns.

Retirement, as a major life transition, involves substantial restructuring of use of time, and as such offers both risks and opportunities for health. Previous work suggests that retirement may be an effective intervention point for changing health behaviours [38]. The results obtained from the Life After Work study have the potential to inform the development of lifestyle interventions to assist people with healthy and satisfying retirement. For example, just as seminars and counselling are widely available to assist pre-retirees plan financially for their retirement, findings could be used to develop programs to assist pre-retirees to plan how they will be physically, socially and mentally active in retirement, in order to optimise health and wellbeing. The study may also identify problematic behaviours, such as a decrease in social interaction or an increase in sedentary behaviour, which may warrant specific interventions.

## Competing interests

The authors declare that they have no competing interests.

## Authors’ contributions

TO conceived the study. TO, WB, CM, JVU and NB designed the study. CM oversees the study, deals with multisite issues, and leads recruitment efforts at the South Australian site. JS oversees data collection at the South Australian site, and coordinates the MARCA interviews and data auditing. NB oversees recruitment and data collection at the Queensland site. All authors read and approved the final manuscript.

## Funding

This study and Carol Maher’s Post-Doctoral Fellowship are partially funded by an Australian Research Council Discovery Project Grant DP110101738. The Brisbane component is also supported by a program grant from the (Australian) National Health and Medical Research Council (ID 569940).

## Pre-publication history

The pre-publication history for this paper can be accessed here:

http://www.biomedcentral.com/1471-2458/13/952/prepub
